# Skin Lesion Segmentation and Multiclass Classification Using Deep Learning Features and Improved Moth Flame Optimization

**DOI:** 10.3390/diagnostics11050811

**Published:** 2021-04-29

**Authors:** Muhammad Attique Khan, Muhammad Sharif, Tallha Akram, Robertas Damaševičius, Rytis Maskeliūnas

**Affiliations:** 1 Department of Computer Science, Wah Campus, COMSATS University Islamabad, Wah Cantonment 47040, Pakistan; sharif@ciitwah.edu.pk; 2 Department of Electrical Engineering, Wah Campus, COMSATS University Islamabad, Islamabad 45550, Pakistan; tallha@ciitwah.edu.pk; 3 Faculty of Applied Mathematics, Silesian University of Technology, 44-100 Gliwice, Poland; 4 Department of Applied Informatics, Vytautas Magnus University, 44404 Kaunas, Lithuania; rytis.maskeliunas@vdu.lt

**Keywords:** skin cancer, melanoma, heuristic feature optimization, moth flame optimization, deep features, feature fusion

## Abstract

Manual diagnosis of skin cancer is time-consuming and expensive; therefore, it is essential to develop automated diagnostics methods with the ability to classify multiclass skin lesions with greater accuracy. We propose a fully automated approach for multiclass skin lesion segmentation and classification by using the most discriminant deep features. First, the input images are initially enhanced using local color-controlled histogram intensity values (LCcHIV). Next, saliency is estimated using a novel Deep Saliency Segmentation method, which uses a custom convolutional neural network (CNN) of ten layers. The generated heat map is converted into a binary image using a thresholding function. Next, the segmented color lesion images are used for feature extraction by a deep pre-trained CNN model. To avoid the curse of dimensionality, we implement an improved moth flame optimization (IMFO) algorithm to select the most discriminant features. The resultant features are fused using a multiset maximum correlation analysis (MMCA) and classified using the Kernel Extreme Learning Machine (KELM) classifier. The segmentation performance of the proposed methodology is analyzed on ISBI 2016, ISBI 2017, ISIC 2018, and PH2 datasets, achieving an accuracy of 95.38%, 95.79%, 92.69%, and 98.70%, respectively. The classification performance is evaluated on the HAM10000 dataset and achieved an accuracy of 90.67%. To prove the effectiveness of the proposed methods, we present a comparison with the state-of-the-art techniques.

## 1. Introduction

According to the World Health Organization (WHO), skin cancer accounts for one-third of all types of cancers [1,2]. Each year, skin cancer cases increase and result in a higher number of deaths. Currently, approximately 3 million non-melanoma and 132,000 melanoma skin cancer cases are diagnosed globally every year [3]. According to the WHO, 9500 people receive a skin cancer diagnosis every day in the US alone, and two people die every hour [4]. The average annual cost of treating such cases is USD 3.3 and 4.8, respectively [5]. As per the statistics, the number of invasive melanoma cases has increased by 47% in the last ten years [6]. In Europe, more than 100,000 new cases of diagnosed melanoma are reported annually. In Australia, on the other hand, the number of annual reported cases of melanoma is 15,229. However, the latest statistics show that the number of skin cancer cases has been increasing since 1990. The current trend was explained by the reducing ozone layer and the increased use of solariums and tanning beds [7].

The biopsy method has been used in practice for examining skin cancers for a few decades now. It is the simplest method available, but its reliability is questionable. Some of the other screening methods include the ABCDE rule [8] and the seven-point checklist [9]. However, these methods require an expert dermatologist. In recent years, dermatologists have used dermoscopy and microscopic images for diagnosing skin cancer [10]. The microscopic images have a low resolution and can be seen through mobile cameras. Dermoscopy, on the other hand, is a new imaging technology that improves diagnostic accuracy and can assist in reducing the human mortality rate [11]. The images captured through dermoscopy are of a high resolution and show deeper skin structures [12]. Expert dermatologists analyze these images through visual inspection. This process requires skill and attention and is time consuming [13]. Computer-aided diagnostics (CAD) systems bypass these problems, consume little time, and provide much better accuracy than these manual techniques [14,15]. CAD systems’ history is not very impressive because they work in several interlocked steps, including preprocessing, handcrafted feature extraction, and then classification, which tend to constrain the overall classification accuracy (OA). These methods initially remove bubbles and artifacts and then perform the next task. In contrast, deep learning methods are superior to these techniques and manual examination. Convolutional neural networks (CNNs) with multiple convolutional layers are typically used in deep learning feature extraction [16,17].

A CAD system performs skin lesion detection using a segmentation approach and skin lesion type classification. The lesion area’s location is detected using any segmentation or deep CNN approach in the first phase. In contrast, in the second phase, the extracted lesion needs to be classified in the relevant category, such as nevi, benign, melanoma, etc. Both tasks, however, are challenging due to the following reasons: (i) the color difference among healthy and lesion regions is not very strong, and sometimes, they both fall in the same category; (ii) the low contrast of skin lesions makes correct detection hard for the segmentation algorithm, and (iii) there are visual differences among the intra type skin lesions. Many techniques have been proposed to resolve these issues, but they could not achieve significant accuracy [18,19]. Skin cancer can be classified into numerous classes; however, only the benign and malignant categories are required in clinical tasks. Typically, benign and malignant lesions have many differences, and binary classification has a higher accuracy rate [20]. It is believed that if one merges several skin lesion classes, the problem of data augmentation could be resolved. However, in some cases, an overfitting problem is observed, which researchers resolve using feature selection techniques along with fitness functions such as KNN [21]. The latter are categorized into filter-based and wrapper-based techniques. The filter-based techniques are much faster than the wrapper-based ones, but the latter are still considered better for various reasons.

Recently, feature fusion and the best feature selection techniques showed an improved accuracy. Many fusion and selection techniques have been introduced in the literature. Most feature fusion techniques follow serial-based approaches and parallel-based approaches. The main purpose of the fusion techniques is to increase the information of an object available from multiple sources. However, this step increases the number of predictors, which has an impact on the computational time. Therefore, researchers of computer vision introduced feature selection techniques for the selection of the best features. Feature selection techniques are categorized as heuristic-based techniques and meta-heuristic approaches. Meta-heuristic techniques are more useful based on the selection process and have a smaller number of predictors [22].

In this work, we use the HAM10000 dataset [23], which includes seven classes: basal cell carcinoma (BCC), dermatofibroma (DF), melanoma (MEL), benign keratosis (BKL), melanocytic nevi (NV), vascular (VASC), and actinic keratoses (AKIEC). The images in this dataset are not balanced; therefore, it is a great challenge to train a CNN model with it. This dataset includes 15 different attributes, such as hand, face, neck, foot, etc. Each class consists of a different number of images. In the segmentation task, we use the ISBI 2016 [24], ISBI 2017 [25], ISBI 2018 [26], and PH2 datasets [27]. The key challenges while using these datasets are the low-contrast lesions, high irregularity, and lesions on the boundary area.

This article presents a methodology for the segmentation and multiclass classification of skin lesion images and contributes with several new methods as follows:

- For skin image enhancement, we propose a novel hybrid contrast stretching approach, named local color-controlled histogram intensity values (LCcHIV). The method increases the local contrast of a lesion region based on the color and histogram intensity values.
- For segmentation of the skin lesion regions, we propose a new deep learning-based saliency approach, which is implemented using a custom 10-layer CNN. Saliency maps are obtained by fusing 128 channels of the third convolutional layer of the CNN. The fusion of these channels returns a saliency map, which is further converted into binary using a thresholding function. Additionally, an active contour-based mask is generated for localization of the segmented lesions.
- For deep feature extraction, we use transfer learning from two pre-trained CNN models (ResNet101 [28] and DenseNet201 [29]), where the most important features are selected using an improved moth flame optimization (IMFO) algorithm.
- Finally, we adopt a multiset maximum correlation analysis (MMCA) approach for feature fusion, while the classification of fused features is performed using the Kernel Extreme Learning Machine (KELM).

The rest of the manuscript is organized as follows: the proposed methodology is explained in Section 2. The proposed method is presented in Section 3, with a detailed mathematical explanation and visual results. Section 4 discusses the experimental setup and results. Finally, the conclusions are given in Section 5.

## 2. Related Works

Several skin lesion segmentation and classification techniques exist in the literature—using either conventional or deep methods. For example, Khan et al. [30] presented a novel technique based on probabilistic distribution and feature selection for skin lesion detection and classification. Normal and uniform distributions are implemented to segment the lesion area. Later, the features are extracted from the segmented images, which are finally fused using a parallel fusion strategy. For feature selection, the entropy-based technique is combined with Bhattacharyya distance and variance formulation. The proposed technique is evaluated on three publicly available datasets, including combined ISBI 2016 and ISBI 2017, ISIC, and PH2, achieving an accuracy of 93.2%, 97.75%, and 97.5%, respectively.

Tschandl et al. [31] trained a fully convolutional neural network (CNN) on the ISIC 2017 dataset and reused the ResNet34 layers to segment skin lesions. Pre-training and fine tuning of ResNet34 improves the segmentation performance; therefore, the mentioned steps were embedded.

Mahbod et al. [32] explored the image resizing effect on pre-trained CNN models to classify skin lesions. The images were resized on six different scales to investigate the classification results of three CNN architectures, namely SeReNeXt-50, EfficientNetB0, and EfficientNetB1. They also developed and evaluated a multi-scale multi-CNN (MSM-CNN) fusion technique based on the ensemble method. This approach utilized the three CNN models trained on cropped images of different sizes. The MSM-CNN technique achieved an 86.2% accuracy on the ISIC 2018 dataset. They also concluded that image cropping yields better results as compared to image resizing.

To classify the skin lesion images into seven classes, the researchers in [33] proposed a deep CNN architecture. They implemented GoogleNet and Inception-V3 to perform binary classification. They improved the accuracy by up to 7% for the multiclass problem.

Chaturvedi et al. [34] presented an automated classification system for multiclass skin cancer. They performed extensive experiments on pre-trained CNN models including Xception, NASNetLarge, Inception-V3, InceptionResNet-V2, and ResNetXt-101 and the ensembles of these models. These CNN architectures were fine-tuned on seven classes of the HAM10000 dataset using transfer learning. This proposed model achieved an accuracy of 93.2% on the ResNetXt-101 model. The accuracy achieved on the ensemble of InceptionResNet-V2 and ResNetXt-101 was 92.83%.

Al-Masni et al. [35] presented a hybrid model for multiple skin lesion classification and segmentation. A full-resolution convolutional network (FrCN) was utilized for segmentation of lesion parts. Deep CNN classification performed on the segmented skin lesions. The presented technique was validated on three challenging skin datasets, ISIC 2016, ISIC 2017, and ISIC 2018, with proper data normalization of these datasets and achieved improved results.

Xie et al. [36] presented a mutual bootstrapping deep convolutional neural network (MB-DCNN) for efficient detection and classification of skin lesions. A coarse segmentation network was utilized for enhanced segmentation of lesions and a mask-guided network was implemented for classification. Bootstrapping coarse segmentation networks and enhanced segmentation networks played a vital role in segmentation and classification. The features of both networks were concatenated for efficient detection and classification. The proposed technique was validated on the ISIC 2017 and PH2 datasets and achieved mean area under the curve (AUC) values of 93.8% and 97.7%, respectively.

Jayapariya et al. [37] introduced a fully convolutional network-based model for melanoma detection. The VGG16 [38] and GoogleNet [39] deep CNN models were used for segmentation of lesions, followed by the feature extraction step. The deep CNN-extracted features were fused for accurate segmentation. Later, they extracted handcrafted features and concatenated them with a deep vector. The SVM was added at the end for a final classification. The presented model was tested on the challenging ISIC 2016 and ISIC 2017 datasets, with an accuracy of 0.8892 and 0.853.

Xie et al. [40] focused on spatial features for lesions’ segmentation by introducing a high-resolution CNN model. In this model, they extracted deep features without affecting the spatial attributes and decreasing the noise effect. The proposed model robustly segmented the lesions by overcoming the artifacts and hair distraction.

Miglani et al. [41] compared the performance of deep CNN models for the robust classification of skin lesions. Transfer learning was performed using ResNet-50 [28] and EfficientNet-Bo by fine-tuning their parameters. The HAM1000 dataset was utilized to validate the performance of the deep CNNs. EfficientNet-Bo outperformed ResNet-50 by achieving macro and micro AUC value of 0.93 and 0.97 for skin lesion classification.

Mahbod et al. [42] proposed using multiple pre-trained CNNs with different architectures that are fine-tuned on dermoscopic skin lesion images. The deep features acquired from each CNN were used to train different SVM classifiers. Finally, the prediction probability classification vectors were fused to provide a final prediction. The proposed method achieved an 87.3% AUC using the skin lesion images from the ISIC 2017 dataset. Finally, Mahbod et al. [43] analyzed the impact of various segmentation masks but observed no significant difference between using manually or automatically created segmentation masks on the images from the ISIC 2017 dataset.

In brief, the discussed techniques are mostly based on pre-trained CNN models for feature extraction. Additionally, they used CNN segmentation models for lesion detection. As mentioned earlier, the studies’ primary focus was to improve the accuracy of a system; however, they did not focus on the system’s prediction time. In this work, we select the optimal deep features and combine features based on the correlation-related fusion approach. Most of the above-discussed articles used the SVM for the final classification stage, but our target is a multiclass learner, and therefore, we utilized the Kernel Extreme Learning Machine (KELM).

## 3. Materials and Methods

This section discusses the benchmark datasets and the pre-trained models utilized in the framework. To authenticate the proposed frameworks, results were generated from four datasets including ISBI 2016, ISBI 2016, ISBI 2017, ISIC 2018, and PH2 and HAM10000. Similarly, two pre-trained models, namely ResNet101 [28] and DenseNet201 [29], were utilized for feature extraction.

### 3.1. Datasets

The following datasets were used in this work for evaluation of the proposed method.

**Figure 1:**
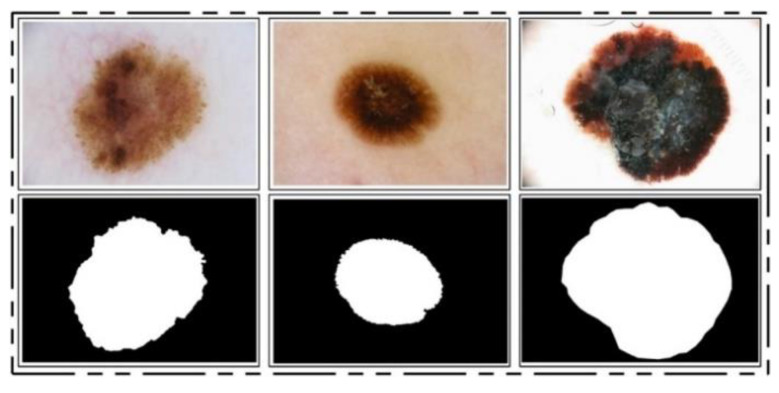
Sample images of ISBI 2016 for skin lesion segmentation.

ISBI 2016 Dataset: This dataset [24] entails a total of 900 training images and 379 testing images. The ground truth images are publicly available for validation of the segmentation task. The training images include melanoma and benign classes. These images were used for the training of a CNN model for the lesion segmentation task, while the rest of the testing images were utilized for testing the newly implemented CNN model for the segmentation task. Some sample images are reproduced in Figure 1.

**Figure 2:**
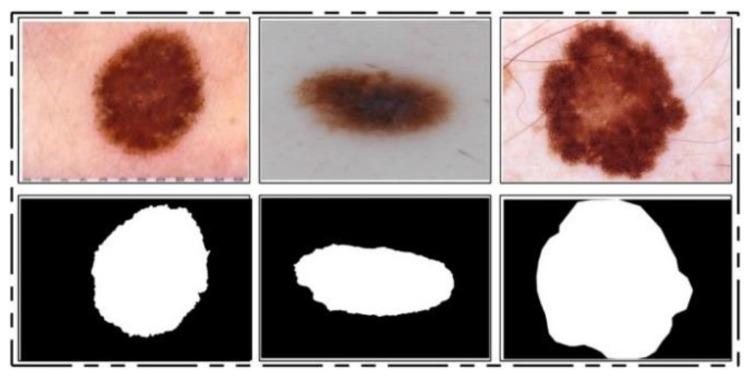
Sample images of the ISIC 2017 dataset.

ISIC 2017 Dataset: This dataset [25] consists of a total of 2750 dermoscopy images. From those, 2000 images are used for training, 150 for validation, and 600 for testing [25]. The ground truth samples of this dataset are also publicly available, which were used for validation of the segmentation algorithm. A few sample images are shown in Figure 2. For the classification task, images were classified into three categories, namely melanoma (374), seborrheic keratosis (254), and nevi (1372). As these classes are not balanced, we performed data augmentation.

**Figure 3:**
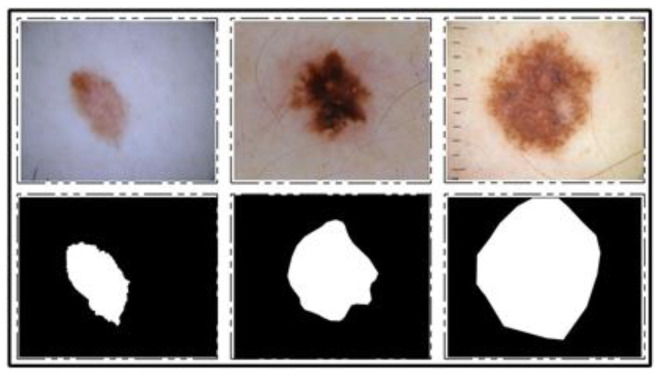
Sample images for lesion segmentation task from ISIC 2018 challenge dataset [26].

ISBI 2018 Dataset: This dataset consists of three parts—lesion segmentation, attribute detection, and classification of the lesion into type [26]. For segmentation, a total of 2594 training images are provided along with ground truth images, whereas 1000 and 100 images are given for testing and validation, respectively. A few sample images along with ground truth images are shown in Figure 3.

**Figure 4:**
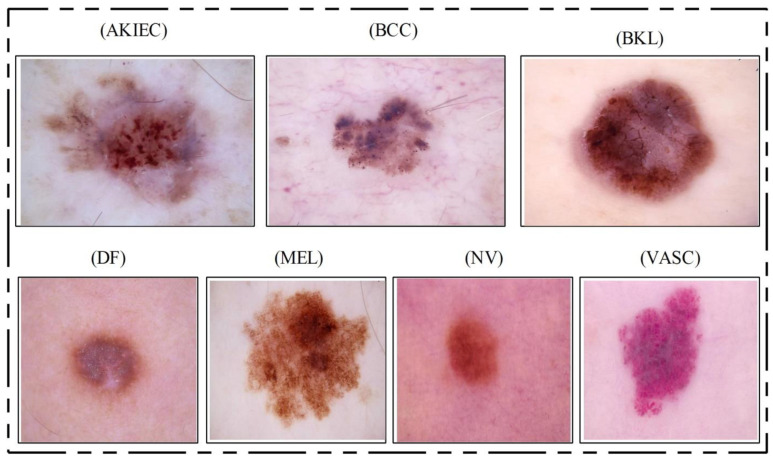
Sample skin lesion types collected from the HAM10000 dataset [23].

HAM10000 Dataset: This dataset [23] includes a total of 10,015 dermoscopy images. This dataset is known as one of the most complex imaging databases for multiclass skin lesion classification. Seven different types of skin lesions are included, namely AKIEC, BCC, BKL, DF, NV, MEL, and VASC. For each label, the number of images included is 327, 541, 1099, 155, 6705, 1113, and 142, respectively. Figure 4 shows few sample images from this dataset.

### 3.2. Proposed Framework

A fully automated system is proposed for lesion segmentation and multiclass classification using optimal deep learning features. The proposed framework incorporates five primary steps: contrast stretching by implementing a hybrid approach named local color-controlled histogram intensity values (LCcHIV). Later on, a deep saliency-based technique is proposed, which initially computes the saliency map, which is refined through the superpixel technique. After that, images are converted into binary form and morphological operations are performed for refinement of the extracted lesions. In the third step, the ResNet101 and DenseNet201 pre-trained networks are implemented and trained through transfer learning (TL).

**Figure 5:**
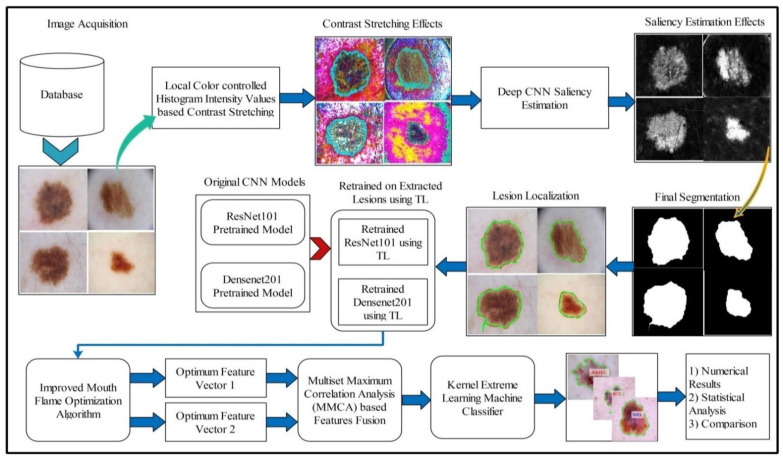
Proposed systematic diagram of multiclass skin lesion localization and classification.

Features extracted from both models are optimized through an improved MFO algorithm. This algorithm is separately applied on both extracted vectors, and resultant vectors are fused using correlation analysis (CA). Finally, the fused vector is classified using the KELM classifier and the results are evaluated on selected datasets. A detailed systematic flow is shown in Figure 5 and described below.

### 3.3. Lesion Contrast Stretching

For image quality assessment, contrast enhancement is one of the imperative requirements. Image enhancement is the process of improving image quality by increasing the quality of a few features or decreasing haziness among distinct image pixels. The key objective of this step is to improve the image quality compared to the original image. Our main objective was to enhance the contrast of the lesion region to easily extract the region of interest (ROI). Many techniques are presented in the literature, and one of the most famous techniques is histogram equalization (HE) [44]. In HE, all image pixels are increased, but lower and upper bounds are set to perform the enhancement only in the specific region of the lesion.

Motivated by HE, we implemented a hybrid contrast stretching technique named local color-controlled histogram intensity values (LCcHIV). In this approach, we initially create a histogram of the input image to find the lesion pixels and then improve the pixel range by multiplying variance values. The resultant variance value-based image is subjected to HE for further refinement. Later, the intensity values are increased and adjusted according to the lesion and background regions based on a fitness function. This process is described as follows.

Consider an input image $\xi_{xy}$ having dimensions N×M, where N=512 and M=512, and (xy)∈R. Let ${\tilde{\xi }}_{xy}$ be the resultant contrast-enhanced image of the same dimensions as the input image $\xi_{xy}$. First, the histogram of the image $\xi_{xy}$ is computed as follows:

(1) $$h_{f}\left(k\right)=O_{j}$$

where $h_{f}\left(k\right)$ is the histogram of an image $\xi_{xy}$, f represents the frequency of occurrences, $O_{j}$ represents the occurrence of gray levels, and j∈0,1,2,…K−1. Based on $h_{f}\left(k\right)$, we find the range of infected pixels, represented by Equation (2).

(2) $${\tilde{h}}_{f}\left(k\right)=h_{f}\left(k\right){\left[I_{j}\right]}_{k_{1},k_{n}},$$

where $I_{j}$ represents the infected region patch and j represents the pixel values. The ${\tilde{h}}_{f}\left(k\right)$ is the entire infected region, and the range of the infected region is represented by $k_{1}$ to $k_{n}$. Later, we compute the variance of the whole image $\xi_{xy}$ using Equation (3).

(3) $$\sigma^{2}\left(\xi_{xy}\right)=\frac{1}{MN}\sum_{i=0,\;j=0}^{M-1,N-1}{\left(\xi_{ij}\right)}^{2}-\mu^{2},$$

(4) $$\mu =\frac{\sum_{i=0,\;j=0}^{N-1,\;M-1}\left(\xi_{ij}\right)}{MN}$$

The output variance value is multiplied with Equation (2) as follows:

(5) $${\tilde{H}}_{f}\left(k\right)=\left[{\tilde{h}}_{f}\left(k\right)\right]\times \left[\sigma^{2}\left(\xi_{xy}\right)\right]$$

After that, we fuse $\xi_{xy}$ and ${\tilde{H}}_{f}\left(k\right)$ to obtain an infected patch. Furthermore, histogram equalization is applied on the infected patch and fused with the original image in Equation (6).

(6) $$F\left(\xi_{xy},{\tilde{H}}_{f}\left(k\right)\right)={\left[\xi_{xy}\right]}_{N\times M}{\left[{\tilde{H}}_{f}\left(k\right)\right]}_{N\times M}$$

**Figure 6:**
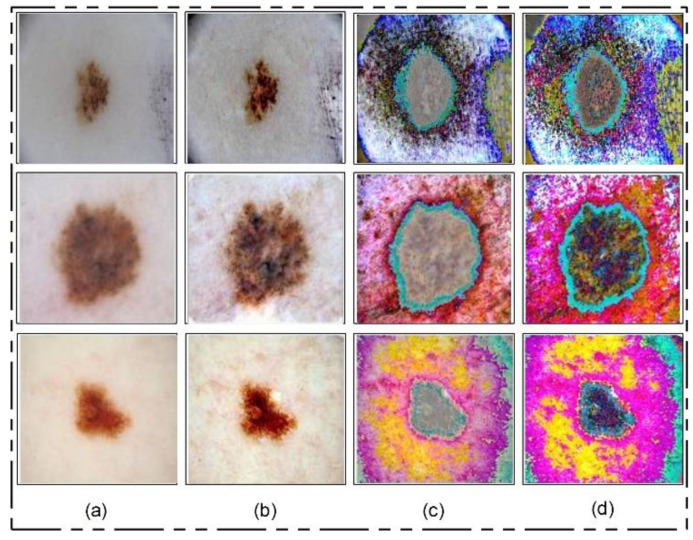
Lesion contrast stretching results. (**a**) Original Image; (**b**) fused image; (**c**) output of first fitness function; (**d**) output of second fitness function.

The process results are visually shown in Figure 6b. In this figure, it is shown that the infection regions are highlighted more as compared to the original images. However, our interest is to separate the infection part from background based on contrast. Therefore, we define two fitness functions in sequential order and obtain more relevant information. Mathematically, the fitness functions are defined as follows:

(7) $$Fitness\left(1\right)=↑\left[F\left(\xi_{xy},{\tilde{H}}_{f}\left(k\right)\right)\right]+L_{\left(5\right)}$$

(8) $$Fitness\left(2\right)=↑\left[Fitness\left(1\right)\right]+L_{\left(5\right)}$$

These functions increase the range of pixel values by up to 5 times, each time in an increment of 1. After the images are passed through both fitness functions, the resultant images are more informative for correct lesion segmentation. The visual results of this step are shown in Figure 6c,d. The final result shown in Figure 6d is utilized in the next step for lesion segmentation.

### 3.4. Deep Saliency-Based Lesion Segmentation

Segmentation of skin lesions is a crucial step for the localization of infected regions. Many techniques are presented in the literature for lesion segmentation using saliency techniques and convolutional neural network-based techniques, to name a few [40,45,46]. The saliency-based techniques are simple, but not more accurate as compared to CNN-based techniques. The techniques based on CNN [47,48] required a large number of ground truth images for training a model, which is further utilized for the detection process. However, in most medical applications, it is not possible to prepare the required number of ground truth images (e.g., for skin cancer, stomach infections, COVID-19, and lung cancer). Moreover, the existing techniques are also facing a few problems including low contrast of lesions, complex lesion boundaries, and irregularity in lesion shapes [49]. We propose a new method named Deep Saliency Segmentation (DSS) for skin lesion detection in this work. The proposed method works as follows: (i) a simple CNN model is designed, which includes ten layers; (ii) features of the last convolutional layer are visualized and concatenated in one image; (iii) superpixels of the concatenated image are computed; (iv) a threshold is applied for the final segmentation; and (v) boundaries are drawn on segmented regions using an active contour approach for the localization of skin lesions.

**Figure 7:**
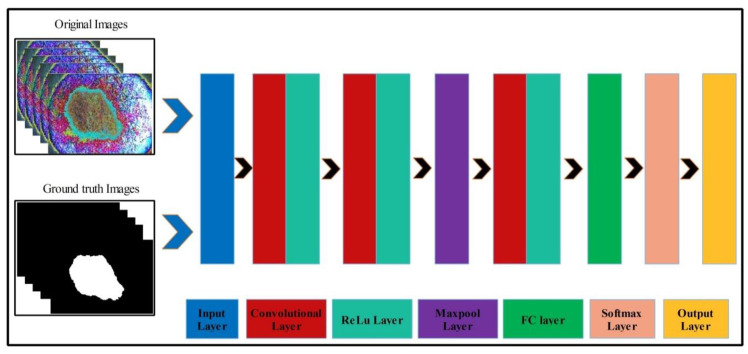
Designed CNN model for skin lesion visualization.

Mathematically, this approach is formulated as follows: Given that ${\tilde{\xi }}_{xy}\in Fitness\left(2\right)$ denotes output enhanced images with dimensions of 512×512×3, we utilized these images to design a simple CNN model. The main use of this model is to learn and visualize the features of an image. Visually, the designed model is shown in Figure 7. This model includes several layers, such as one input layer, three convolutional layers along with the ReLu layer, one max-pool layer, one fully connected layer (FC), one softmax layer, and, finally, an output layer. The size of the input layer was 224×224×3; therefore, we resized all images to this size. In the first convolutional layer, the filter size was [3,3], the number of channels was 3, the filter size was 64, and stride was [1,1]. After this layer, we obtained two feature matrices named the weight matrix and the bias matrix. The size of the weight matrix was 3×3×3×64, and the bias matrix was 1×1×64.

The convolutional layer weight matrix and bias matrix are represented as follows:

(9) $$C_{h}^{L}=\sum_{h}{\tilde{\xi }}_{xy}\times W_{h}^{L}+b_{h}^{L},$$

where $C_{h}^{L}$ denotes features of the first convolutional layer, ${\tilde{\xi }}_{xy}$ is an enhanced image, $W_{h}^{L}$ is the weight matrix of the lth layer, and $b_{h}^{L}$ is the bias matrix of the lth layer. After that, the ReLu activation layer was applied. In the second convolutional layer, the filter size was [3,3], the number of channels was 64, the number of filters was 64, and stride was [1,1]. The weights and bias of this layer were updated as follows:

(10) $$W_{h}^{L}\left(i+1\right)=\frac{-r}{q}W_{h}^{L}-\frac{r}{N}\left(\frac{\partial F}{\partial W}\right)+\tilde{m}\psi W_{h}^{L}\left(i\right),$$

(11) $$b_{h}^{L}\left(i+1\right)=-\frac{r}{N}\left(\frac{\partial F}{b_{h}^{L}}\right)+\tilde{m}b_{h}^{L}\left(i\right),$$

where $W_{h}^{L}\left(i+1\right)$ is the updated weight matrix, $b_{h}^{L}\left(i+1\right)$ is the updated bias matrix, r is the learning rate, and $\tilde{m}$ is the momentum. The features of this layer were normalized using the ReLu activation function. Next, a max-pooling layer was applied of filter size [2,2] and stride of [2,2]. The main purpose of this layer was to obtain more active features and minimize the feature length. Mathematically, this layer is formulated as follows:

(12) $$M_{h}^{L}\left(W\right)=max\left\{W_{h}^{L}\left(i+1\right)\right\},$$

(13) $$M_{h}^{L}\left(b\right)=max\left\{b_{h}^{L}\left(i+1\right)\right\}$$

In the third convolutional layer, the filter size was [3,3], the number of channels was 64, the number of filters was 128, and the stride was [1,1]. The weights and the bias matrix were updated using Equations (10) and (11). Later, the fully connected and softmax layers were added and training was performed.

**Figure 8:**
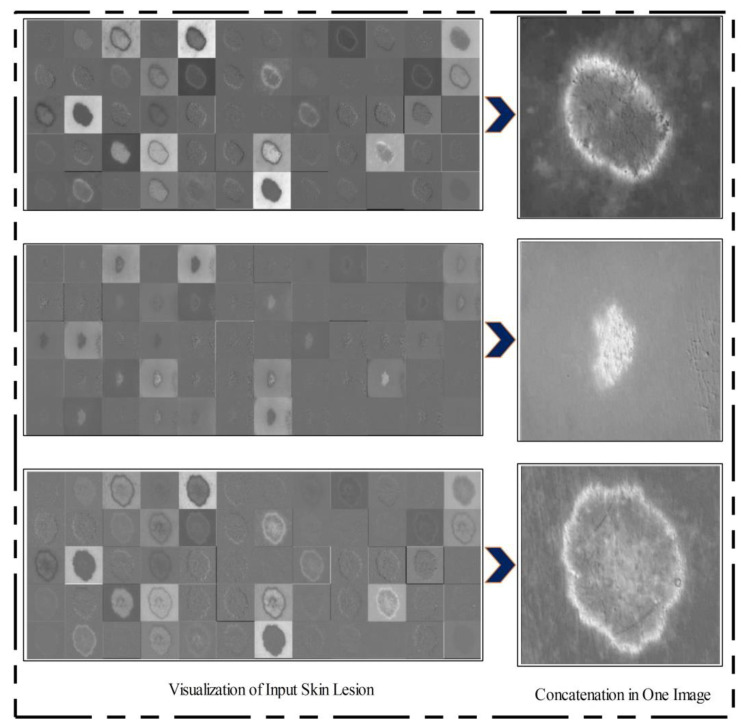
Feature visualization of the third convolutional layer.

**Figure 9:**
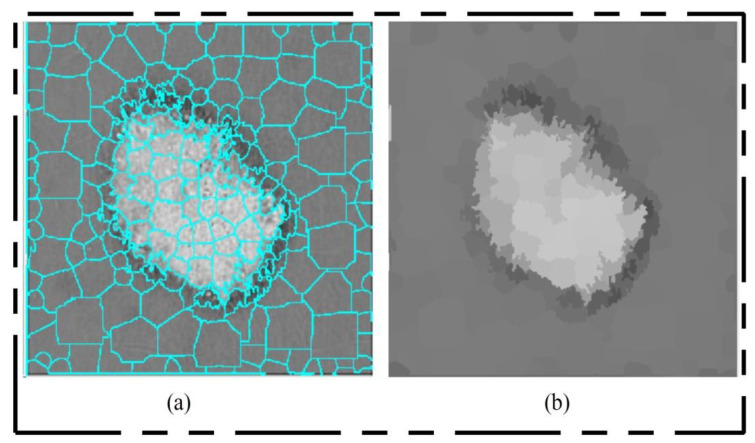
Superpixel generation. (**a**) Superpixel boundaries; (**b**) mean superpixel-based infected region.

After training, the features of the third convolutional layer were visualized, and in the output, 96 images were generated, as shown in Figure 8. Then, all 96 output images were combined in one image to obtain a saliency map image. Visually, this resultant image is shown in Figure 8. This figure shows that the output image is more informative, and the lesion is highlighted. By utilizing this saliency map image, superpixels were computed and the pixels were reconstructed. The main purpose of the superpixel technique is to group the important image pixels of a meaningful object. The second purpose of this approach is to remove image complexity. In this work, we used a simple linear iterative clustering approach [50] for superpixel generation. This approach is shown in Figure 9. This figure illustrates that initially, superpixels are generated, and then, they are combined based on color pixels.

After this technique, the constructed saliency map was clearer, which was further passed through a threshold function for final segmentation. Mathematically, the threshold function is defined as follows. Consider that ${\tilde{\xi }}_{sal}\left(x,y\right)$ is a final saliency mapped image and τ represents a threshold value; then, the threshold function is formulated as follows:

(14) $$\tau =\mu \left({\tilde{\xi }}_{sal}\left(x,y\right)\right),$$

(15) $${\tilde{\xi }}_{seg}\left(x,y\right)=\{\begin{array}{c} 1\;\;for\;{\tilde{\xi }}_{sal}\left(x,y\right)>\tau \\ 0\;\;for\;{\tilde{\xi }}_{sal}\left(x,y\right)\leq \tau \end{array}$$

**Figure 10:**
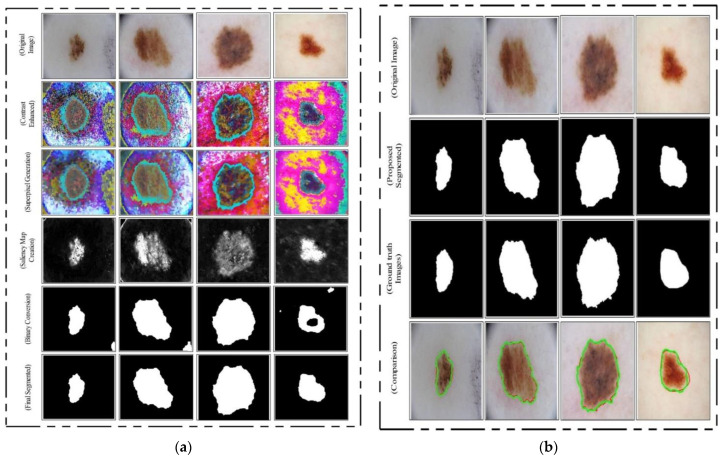
Proposed skin lesion segmentation results. (**a**) Intermediate segmentation results, (**b**) segmentation results compared with ground truth images.

This threshold function converts images into binary format. Visually, the output is shown in Figure 10 (binary image), which illustrates that the binary images include a few holes that need to be refined. Therefore, we applied a filling morphological operation. The final segmented images are shown in Figure 10a. We drew boundaries based on the active contour approach (seen in Figure 10b). This figure illustrates that the boundaries drawn on the original images are based on their segmented output. Hence, in the next step, deep learning features were extracted through these localized regions.

### 3.5. Multiclass Lesion Classification

Multiclass skin lesion classification is a new research area in which researchers are trying to improve the classification performance. Experiments have been performed on the ISBI 2018 and HAM10000 datasets. However, the images in these datasets are very similar to each other, and each dataset consists of seven classes, as mentioned in Section 3.1. In the existing studies, the researchers faced high similarity, low contrast, and imbalanced data. In this work, for the multiclass classification, we initially balanced the data using a data augmentation step. For this purpose, we performed the following operations: right flip, left flip, and transposition of the original and both flipped images. After balancing the skin classes, we utilized two pre-trained deep learning models named ResNet101 and DenseNet201. We retrained both models by employing transfer learning for feature extraction. A detailed description is given below.

**Figure 11:**
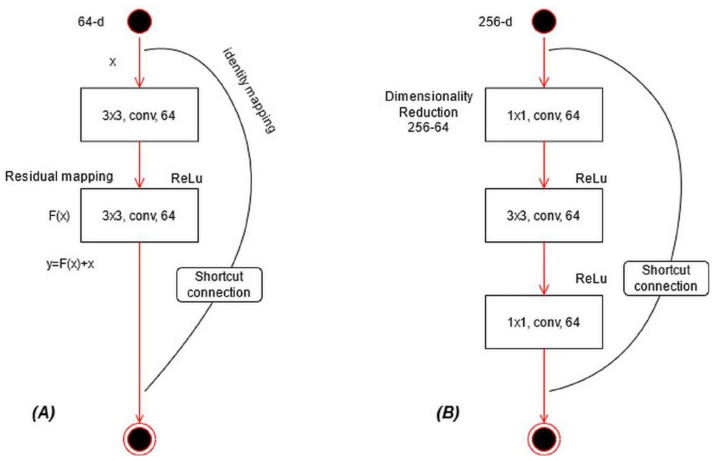
Type of building blocks for ResNet: (**A**) a basic building block, (**B**) a bottleneck block.

**Figure 12:**
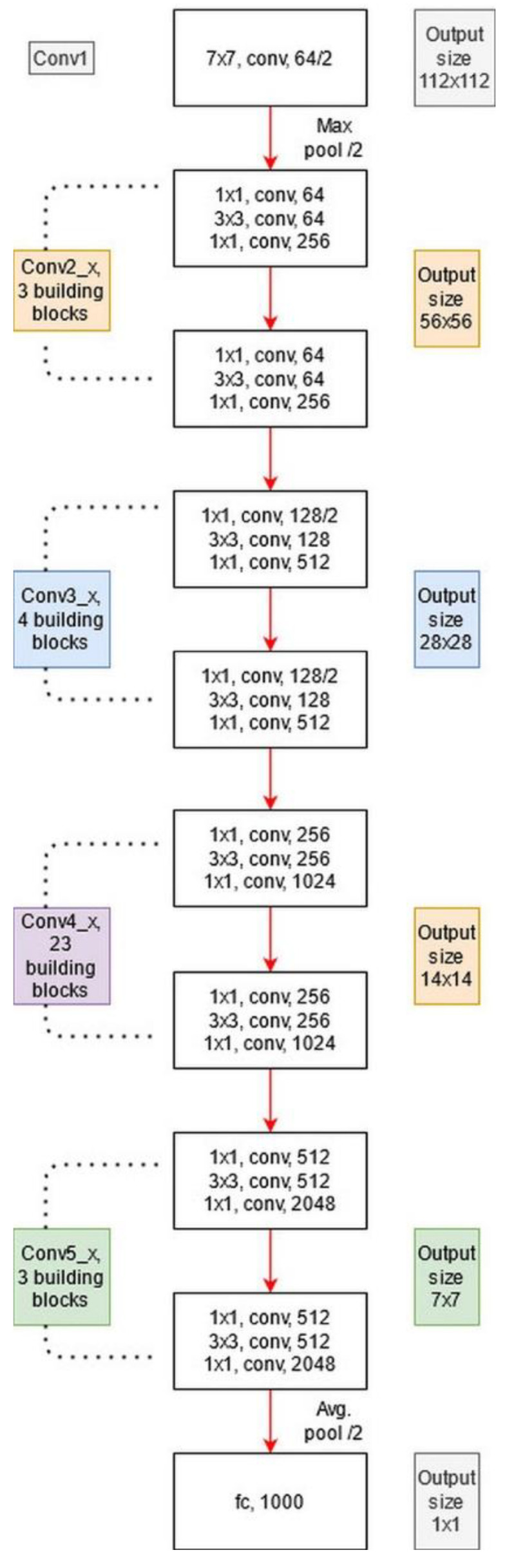
Architecture of ResNet101 pre-trained CNN.

ResNet101 CNN Model: ResNet was proposed in 2015 [28]; see Figure 11A. In this model, a few connections are simply skipped, and the direct connections are made between the layers. In ResNet101, the “bottleneck” building blocks are used to reduce the parameters (see Figure 11B). These blocks are formulated as follows:

(16) $$\hat{Y}=R\left({\tilde{\xi }}_{loc},\left\{w_{j}\right\}\right)+{\tilde{\xi }}_{loc}$$

where $\hat{Y}$ represents an output vector, R(.) is the residual mapping to be learned, and ${\tilde{\xi }}_{loc}$ is input localized lesion pixels. This equation is utilized for short connections, but the dimensions of input and output must be the same. However, if the input–output channels do not have the same dimension, then linear projection is performed. Mathematically, the linear projection is defined as follows:

(17) $$\hat{Y}=R\left({\tilde{\xi }}_{loc},\left\{w_{j}\right\}\right)+\phi_{s}\left({\tilde{\xi }}_{loc}\right),$$

where $\phi_{s}\left({\tilde{\xi }}_{loc}\right)$ represents a convolutional operation, which is utilized to adjust the input dimensions. Visually, the architecture of ResNet101 is shown in Figure 12. The network incorporates five convolutional blocks: Conv1, represents the first convolutional layer; Conv2 includes three building blocks, and each block has three convolutional layers. The third convolutional layer includes four building blocks. In the fourth and fifth convolutional layers, there are 23 and 3 building blocks, respectively. Finally, the last layer is the FC layer used for classification.

DenseNet201: The main idea in ResNet101 was skipping the layers revised in the DenseNet architecture [51]. In this architecture, all features are concatenated in sequential order. Mathematically, the concatenation process is defined as follows:

(18) $${\tilde{z}}_{l}={\tilde{\varphi }}_{l}\left(\left[{\tilde{z}}_{0},{\tilde{z}}_{1},\ldots ,\;{\tilde{z}}_{l-1}\right]\right),$$

where ${\tilde{\varphi }}_{l}$ is a nonlinear transform defined as a composite function followed by a ReLu activation function. The convolution operation of 3×3 is $\left[{\tilde{z}}_{0},{\tilde{z}}_{1},\ldots ,\;{\tilde{z}}_{l-1}\right]$, which refers to the concatenated features for layer l−1. In this model, dense blocks are created for downsampling. These dense blocks are separated by the transition layer.

**Figure 13:**
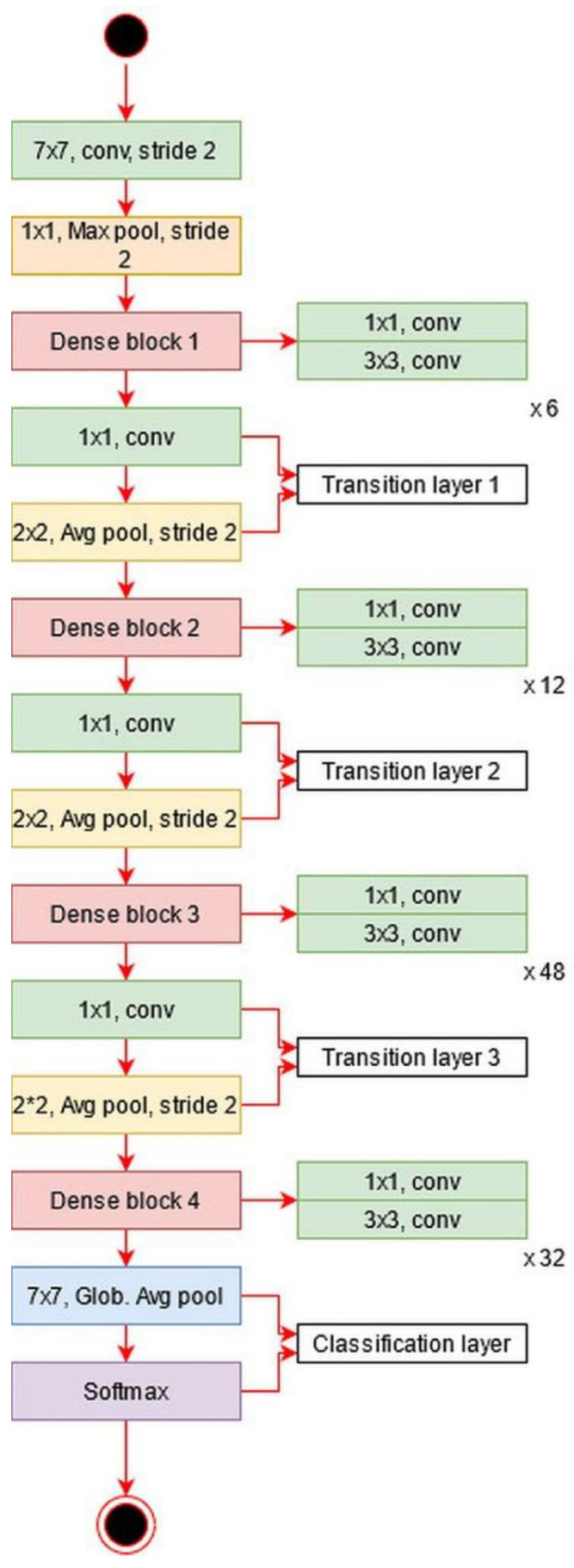
Architecture of DenseNet201 deep learning model.

The architecture of DesnseNet201 is illustrated in Figure 13. This figure describes that the first convolutional layer has a filter size of 7×7 and stride of [2,2]. After this layer, a max-pooling layer is added of a filter size of 3×3. Afterward, a dense block is added and each dense block includes a convolutional layer of filter size 1×1 and 3×3. The main purpose of adding this 1×1 convolutional layer is to reduce the feature map and decrease the computational cost. A total of four dense blocks have been added to this architecture, and for each dense block, convolutional bocks are added. The size of the convolutional blocks is 6, 12, 48, and 32, respectively. After each dense block, a transition layer has been added. After the fourth dense block, a global average pooling layer has been added of a filter size of 7×7, followed by an FC layer for final classification.

**Figure 14:**
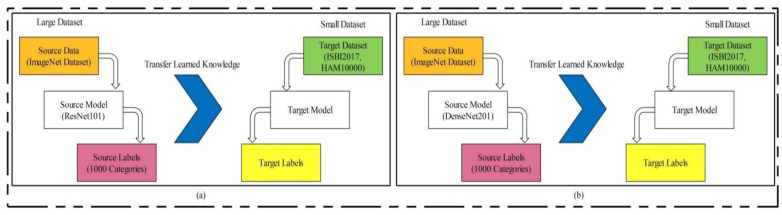
Transfer learning concept for reusing a model for feature extraction: (**a**) using ResNet101 model, (**b**) using DenseNet201 model.

Transfer Learning-based model training: Transfer learning (TL) is a technique where an existing pre-trained model is reused for a new classification task [52]. TL has been proven to achieve good performance on many image classification tasks [53,54,55,56]. Visually, this process is illustrated in Figure 14, where the pre-trained models are trained on large datasets such as ResNet101 and DenseNet201 in this work. In TL, knowledge is transferred on a target model, and the target datasets used were ISBI 2017 and HAM10000. After reusing both models, two new models were obtained for the classification of multiclass skin lesions. In the TL phase, we selected 70% images for training the model and the remaining 30% were used for testing. The number of epochs was 20 and the learning rate was 0.0001. The mini-batch size was set as 64. Mathematically, we can define TL as follows.

Consider that $S_{D}$ is a source domain and $S_{T}$ is a source task; then, it can be defined as $\left\{\left(S_{D},S_{T}\right)|\;i=1,2,3,\ldots ,m^{s}\right\}$. The target domain is denoted by $T_{D}$ and the target source is denoted by $T_{S}$; then, it is represented as $\left\{\left(T_{D},T_{S}\right)|\;j=1,2,3,\ldots ,m^{T}\right\}$. TL uses the knowledge of the source domain and transfer in the target domain based on the following objective function.

(19) $$f^{T_{j}}\left(j=1,2,3,\ldots ,\;m^{T}\right)$$

We selected a global average pool layer of both architectures and extract features. The feature matrix length of ResNet101 was N×2048 and N×2048, denoted by $\vartheta^{v1}$ and $\vartheta^{v2}$, respectively.

Feature Optimization: Feature selection is the removal of irrelevant, noisy, and redundant features from an original feature set. It improves machine learning algorithms’ performance in terms of faster training and ease of interpretation, cuts the complexity if the best features are selected, and reduces overfitting [57].

In this work, we implemented an improved moth flame optimization (IMFO) to select the best features from high-dimensional data. MFO emulates the navigation mechanism of moths in nature [58]. Initially, in this algorithm, a population is assigned, denoted by N, and feasible solutions are assigned $\left(\vartheta_{i},\;i=1,2,3,\ldots N\right)$ with features dimension $\vartheta_{ij},\;\left(j=1,2,3,\ldots ,Dim\right)$. The search technique is defined as follows:

(20) $$\Psi_{mfo}=\left(\Psi_{IP},\Psi_{UP},\Psi_{SP}\right),$$

where $\Psi_{IP}$ denotes an initial phase, $\Psi_{UP}$ is an updated phase, and $\Psi_{SP}$ is the stopping condition. In the initial phase, the population is randomly generated, where N=20. The fitness function is applied to evaluate the features by the following equation.

(21) $$Fitness\left(\vartheta_{i}\right)=r\times Error\left(\vartheta_{i}\right)+\left(1-r\right)\times \left(\frac{\left|\Psi_{i}\right|}{Dim}\right),$$

where $Error\left(\vartheta_{i}\right)$ represents the fitness function. In the Cubic SVM, the selected kernel function is cubic, and the method is one vs. all; r is a random value between [0,1] used to balance the classifier accuracy, $\vartheta_{i}$ denotes input features, and $\Psi_{i}$ denotes selected features in i−th iteration. After this operation, the moths are updated based on the flames, but first, we employ an activation function for first-stage feature selection. The activation function is defined as:

(22) $$\Psi_{i}=\{\begin{array}{c} 1,\;\;\;\;\;\;for\;\frac{1}{1+e^{-\Psi_{i}}}>V\\ 0,\;\;\;\;\;\;Otherwise \end{array},$$

where V is a value obtained after the mean operation of each selected feature set $\Psi_{i}$, and it is updated in each iteration. In our work, the value of is mostly 0.4 to 0.48. In this equation, 1 represents the feature that is selected for the next iteration and is considered in the updated phase, whereas 0 represents the feature that is discarded from the next iteration. Using this process, there is a chance that a few important features will be lost but there is also a high probability of good features being transferred for the next iteration. The features are updated through the following formulation.

(23) $$\Psi_{i}=d_{i}e^{lb}cos\left(2\pi l\right)+Flame_{\left(u\right)}\times w+\left(1-w\right)\times u,$$

where i,u=1,2,…N, $d_{i}=\left|Flame_{\left(u\right)}-\Psi_{i}\right|$, and l=[1,−1]. Based on this expression, the new positions of moths are updated w.r.t flames. Here, l=−1 represents the new closest position to the flame and l=1 represents the farthest position. Later, the updated flames are sorted in descending order, defined by $U_{p}\left(\Psi_{i}\right)$, and the entropy value $H\left(U_{p}\left(\Psi_{i}\right)\right)$ is computed. The activation function is updated for the final selection of features in the first iteration based on the entropy value.

(24) $$\Psi_{i}=\{\begin{array}{c} 1,\;\;for\;\frac{1}{1+e^{-U_{p}\left(\Psi_{i}\right)}}>H\left(U_{p}\left(\Psi_{i}\right)\right)\\ 0,\;\;\;\;\;\;\;\;\;\;Otherwise \end{array}$$

The features passed in this function are again evaluated in the fitness function, and this process continues until the iterations are completed. In this work, the numbers of iterations was set to 100. This algorithm was applied on both deep feature vectors, and in the output, two optimized vectors were obtained. The length of the optimized feature vectors was N×1262 and N×826, denoted by $\Psi_{i}{}^{f1}$ and $\Psi_{j}{}^{f2}$, respectively.

Later on, we fused both vectors and obtained a more informative feature matrix. The main purpose of feature fusion is to improve accuracy, but, on the other side, the computational time increases. For feature fusion, we first found the length of both vectors and then selected the feature vector of the maximum length. Based on the maximum length, we found the entropy of fewer length feature vectors and performed padding. After padding, we computed the correlation among pairs of features and selected only those features for the fused vector that have a maximum correlation. Mathematically, it was formulated as follows.

Given two optimal feature vectors $\Psi_{i}{}^{f1}$ and $\Psi_{j}{}^{f2}$ of dimension N×1262 and N×826, respectively, consider that we have a fused feature vector $\Psi_{k}{}^{fu}$ of dimension $N\times k_{3}$, where $k_{3}$ represents the feature dimension of the fused vector. Initially, the maximum dimensional feature vector is found by employing the following equation:

(25) $$Max_{\left(length\right)}=Max\left(\Psi_{i}{}^{f1},\;\Psi_{j}{}^{f2}\right),$$

**Figure 15:**
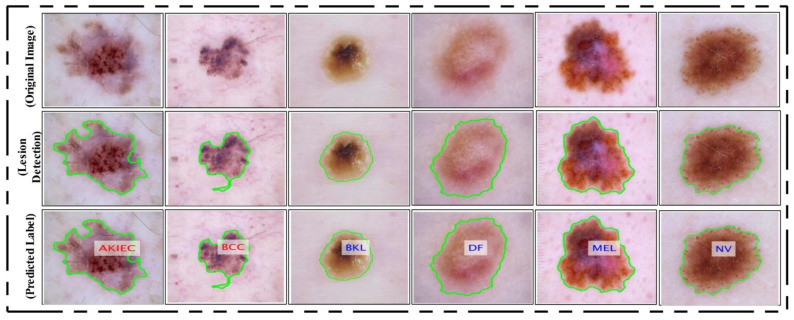
Proposed lesion localization and labeled results. Lesions are labelled as actinic keratosis/Bowen’s disease (intraepithelial carcinoma) (AKIEC), basal cell carcinoma (BCC), benign keratosis (solar lentigo/seborrheic keratosis/lichen planus-like keratosis) (BKL), dermatofibroma (DF), melanoma (MEL), and melanocytic nevus (NV).

This equation returns a maximum length feature vector of dimension $N\times k_{len}$, where $k_{len}=1262$; hence, it is required to equal the length of other feature vectors according to the length of the resultant vector. Mostly, zero padding is performed for an equal length of feature vectors, but we computed the entropy value of fewer feature vectors and performed padding. After this step, the maximal correlation coefficient was computed among pair features (i,j) as follows:

(26) $$\Upsilon \left(i,j\right)=\tilde{\Upsilon }\rho \left(\Psi_{i}{}^{f1},\;\Psi_{j}{}^{f2}\right),$$

(27) $$\rho \left(i,j\right)=\frac{Cov\left(i,j\right)}{\sqrt{\sigma^{2}\left(i\right)}\;\sqrt{\sigma^{2}\left(j\right)}},$$

where Cov(i,j) is the covariance among i and j, $\tilde{\Upsilon }$ is a supremum function [59], $\left(\Psi_{i}{}^{f1},\;\Psi_{j}{}^{f2}\right)\in R$, and the interval is [−1,1], where −1 represents a strong negative correlation among features and 1 represents a strong positive correlation. Hence, we selected only those pair of features that have a maximum correlation (1 or near to 1). Selecting pairs of features through this process plays a key role in obtaining the optimal values. This process of selecting the features was continued until Υ(i,j) was calculated for all feature pairs (1262 pairs). Finally, the resultant fused vector was obtained, and the dimension of $\Psi_{k}{}^{fu}$ was N×1632. This final vector was classified using the KELM [60]. The classification results are shown in Figure 15 as labeled images.

## 4. Experimental Results

### 4.1. Experimental Process

The experimental results of the proposed framework are presented in this section. Results were computed for two different tasks—lesion segmentation and lesion type classification. In the segmentation of skin lesions, two measures were considered—accuracy and error rate. The execution time was also noted for all testing images after the final segmentation. In the classification phase, multiple classifiers were tested to compare the performance of the KELM. These classifiers included Naïve Bayes (NB), Extreme Learning Machine (ELM), multiclass SVM (MSVM), and Fine K-Nearest Neighbor (KNN). Two measures were computed for each classifier—negative rate (FNR) and accuracy. The average classification time was also noted.

### 4.2. Experimental Setup

In this section, we discuss the steps and parameters that were employed during the computation of results. In the skin lesion segmentation process, we tested the implemented CNN architecture. In the testing process, we first trained the model on 50% of the images, which were ground truth images that are publicly available for research purposes. Then, the rest of the images were utilized for testing the implemented CNN segmentation model. In the classification phase, a ratio of 50/50 was employed and 10-fold cross-validation was performed. In the validation phase, several classifiers were used. The first one was Naïve Bayes. In this classifier, the Gaussian function is employed. In the MSVM, the RBF kernel function along with the one vs. all method is employed. In fine KNN, the Euclidean distance method is employed, where the number of neighbors was 10. In the learning process, we opted for a learning rate of 0.001 and a mini-batch size of 28. All simulations of the proposed framework were implemented on a desktop computer with 16 GB RAM and 256 GB SSD. MATLAB 2019b (MathWorks Inc., Natick, MA, USA) was used as a simulation tool. A 16-GB graphics card was also employed to speed up the proposed framework execution time.

### 4.3. Lesion Segmentation Results

**Figure 16:**
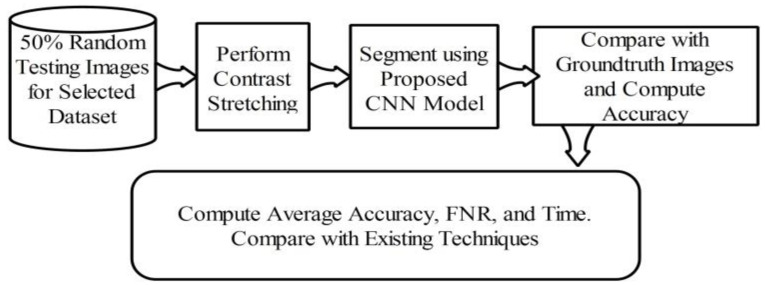
Flow diagram of testing lesion segmentation accuracy using the proposed scheme.

Here, the lesion segmentation numerical results are presented along with visual segmented images. Additionally, a comparison is conducted with recent techniques in terms of accuracy value. Table 1 presents proposed lesion segmentation results for the selected datasets. The results presented in this table were calculated in terms of average accuracy for all selected images of one dataset. Visually, a flow diagram is shown in Figure 16, which shows that original testing images were put in the database and the contrast stretching technique was performed. After segmentation using the newly implemented CNN model, the resultant image was compared with given ground truth images. This process was continued for all images added to the database. After that, the average accuracy, FNR, and overall execution time were obtained for each dataset. As given in Table 1, the average accuracy of the proposed segmentation scheme on ISBI 2016 was 95.38%. The error rate was 4.62% and the testing time of lesion segmentation was 51.3642 (s). Similarly, the accuracy achieved for ISBI 2017 was 95.79% along with an error rate of 4.21%. The noted testing time for this dataset was 59.4160 (s). ISBI 2018 is another challenging dataset, and the achieved accuracy was 92.69%. The error rate and executing time were 7.31% and 67.4003 (s), respectively. Finally, the performance of the PH2 dataset is presented. The achieved accuracy was 98.70% and the error rate was 1.3%. The execution time was 29.3046 (s). Based on the results, it can be observed that the execution time increased according to the size of the dataset. For example, as shown in Table 1, the PH2 dataset, which contained only 100 images, only took 29.3046 (s).

**Figure 17:**
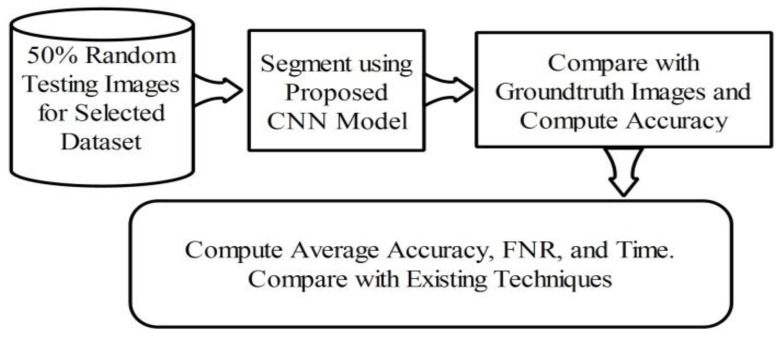
Evaluation of lesion segmentation performance without using contrast stretching step.

To analyze the importance of the contrast stretching step, we also performed segmentation without considering the contrast stretching step. A flow diagram is shown in Figure 17. Note that the contrast stretching step is removed from the original architecture and segmentation is performed. The output results are noted in Table 2. According to this table, the accuracy of ISBI 2016 was 89.37%, which shows that it decreased by an average of 6% as compared to the result in Table 1.

Similarly, for ISIC 2017, ISBI 2018, and PH2, the achieved accuracies were 90.46%, 82.09%, and 91.305, respectively. The change in the results without using the contrast stretching step shows a huge impact on the segmentation accuracy. Overall, an average decrease of 7% in accuracy was shown when we skipped this step for our proposed scheme. Table 3 describes the comparison with existing techniques on the same datasets. According to this table, the previous best noted accuracy on ISBI 2016 was 94.79% [61]. However, our method achieved an accuracy of 95.38% for the same dataset and 95.79%, 92.69%, and 98.70% for the others.

### 4.4. Multiple Skin Lesion Types Classification

Multiple skin lesion classification results are presented in this section. The results were computed using the proposed framework and the resultant values are given in Table 4. The KELM classifier was utilized in the proposed framework, and other classifiers such as Naïve Bayes, ELM, MSVM, and Fine KNN are used for comparison. According to this table, KELM achieved an accuracy of 90.67% and an FNR of 9.33% and the noted time was 133.4406 (s). MSVM achieved the second best accuracy of 85.50% along with an FNR of 85.50% and a time of 121.5200 (s). The time of the MSVM during the testing process was better as compared to the KELM, but there was a large gap among them. The achieved accuracy for Naïve Bayes, ELM, and Fine KNN was 81.34%, 84.92%, and 82.08%, respectively. Table 5 describes the confusion matrix of the KELM. This table can be utilized to verify the proposed accuracy of the KELM. Additionally, in this table, we computed the sensitivity rate of the KELM. The computed sensitivity rate of the KELM was 90.20%. Additionally, this table shows that the skin classes such as BCC, BKL, and MEL had correct prediction rates of 94.60%, 93.04%, and 90.64%, respectively. The worst prediction rate was 84.30% noted for DF.

### 4.5. Analysis

To analyze the classification performance using the proposed framework, we conducted a few experiments:

- Classification performance of ResNet101 using TL;
- Classification performance of DenseNet201 using TL;
- Classification performance using optimal ResNet101 deep features;
- Classification performance using optimal DenseNet201 deep features.

Table 6 describes the classification performance of ResNet101 deep features. The ResNet101 deep features were extracted after TL and showed the best accuracy of 80.46%. MSVM gave the second best accuracy of 77.50%; however, it is noted that only the prediction time for ResNet101 features increased. As presented in Table 4, the best noted time was 121.5200 (s), but in this experiment, the optimal time was 136.3604 (s). The main reason behind an increase in the prediction time is the number of extracted features. Similarly, the classification performance while using only DenseNet201 deep features is given in Table 7. The best accuracy in this experiment was 79.34%, while the worst accuracy was 74.30%. After that, we applied the proposed feature selection approach on each extracted deep feature vector separately, and the results are given in Table 8 and Table 9.

In Table 8, the optimal ResNet101 features are used and achieved an improved performance as compared to the results in Table 6. The best accuracy achieved in this experiment was 83.04% on the KELM classifier, whereas the worst accuracy was achieved by Fine KNN of 76.04%. Additionally, the prediction time was minimized after this experiment due to the reduction in irrelevant features. The best time of this experiment was 96.3248 (s) on MSVM, whereas the KELM was executed in 103 (s).

Similarly, Table 10 describes the classification performance of optimal DenseNet201 features and the attained maximum accuracy of 84.04%. Based on these experiments, it is shown that the optimal solutions provided improved accuracy, but the best time performance was not reached by the proposed framework. This experiment shows the importance of each step based on the results.

**Figure 18:**
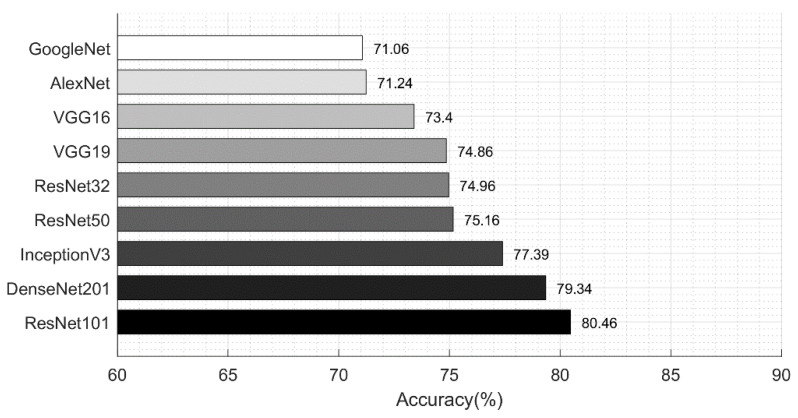
Comparison of neural network accuracy for skin lesion type classification.

Lastly, we also compared the performance of different neural networks to analyze the selection of ResNet101 and DenseNet201. Figure 18 shows the classification performance of various neural networks for the HAM10000 dataset. Neural networks such as VGG16, VGG19, AlexNet, GoogleNet, ResNet32, ResNet50, ResNet101, Inception V3, and DenseNet201 achieved an accuracy of 73.4%, 74.86%, 71.24%, 71.06%, 74.96%, 75.16%, 80.46%, 77.39%, and 79.34%, respectively. These networks were trained on extracted skin lesion types and the results were computed without any feature optimization.

**Figure 19:**
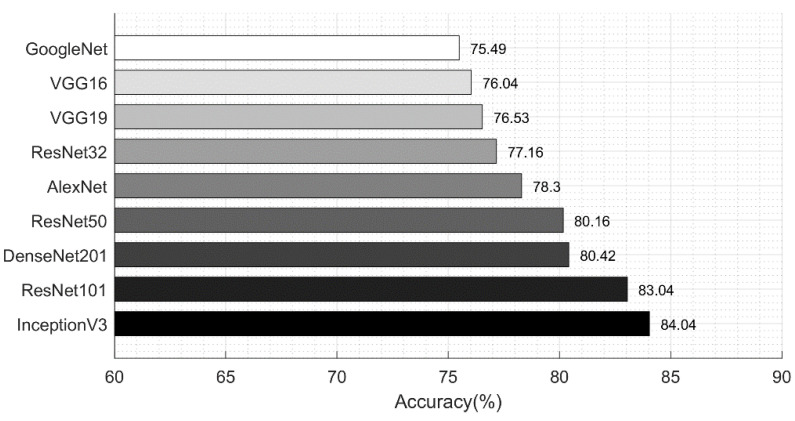
Comparison of neural network accuracy for skin lesion type classification using feature optimization.

Figure 19 shows the classification results achieved after employing the proposed feature optimization approach. The accuracy was improved by an average of 3%, and the computation time was decreased; thus, the optimization of features improves the system performance. We compared these results with those in Table 3, which showed that the proposed optimal feature fusion framework gives a better performance as compared to the individual feature vectors.

A comparison with the previous approaches is given in Table 10. Here, the authors of [64] used the HAM10000 dataset and achieved an accuracy of 83% and a sensitivity rate of 83.0%. In [33], the authors presented an assisted deep learning framework for multiclass classification and achieved a sensitivity rate of 75.57%. The proposed method provided an improved accuracy as compared to existing techniques using the improved MFO (IMFO). Additionally, to support the performance of the IMFO, we computed the results on the original MFO, and the results are given in Table 11. In this case, the original MFO algorithm was added in the proposed flow and the KELM and softmax classifiers were evaluated. Based on the results, the IMFO algorithm increases the classification accuracy. The time complexity of the IMFO algorithm was $O\left(n^{2}\right)$.

## 5. Conclusions

This article proposed a fully automated system for multiclass skin lesion classification. In the framework, a deep learning-based saliency segmentation method and CNN feature optimization using an improved moth flame optimization (IMFO) algorithm were described. The proposed method was evaluated on the well-known ISBI 2016, ISBI 2017, ISBI 2018, and PH2 datasets for the segmentation task and on the HAM10000 dataset for the multiclass skin lesion classification task. The results, upon comparison with the existing methods, show an improved performance. Specifically, the contrast stretching step improves the segmentation accuracy, which is useful for accurate lesion segmentation. Using the segmented lesions, the pre-trained deep learning models used for the extraction of relevant features cannot achieve high segmentation accuracy. To overcome this problem, we removed irrelevant and redundant deep features using an IMFO algorithm, which allowed to achieve improved accuracy. However, one of the constraints in our work is the computational time, which will be addressed in future work. Furthermore, to avoid getting our deep models trained on irrelevant image features, in future work, we will be extending our segmentation technique.

## Figures and Tables

**Table 1:** Accuracy of the proposed lesion segmentation method by employing the contrast enhancement approach.

Dataset	Calculated Measures		
	Accuracy (%)	Error (%)	Testing Time (s)
ISBI 2016	95.38	4.62	51.3642
ISBI 2017	95.79	4.21	59.4160
ISBI 2018	92.69	7.31	67.4003
PH2	98.70	1.3	29.3046

**Table 2:** Lesion segmentation accuracy without employing contrast enhancement approach.

Dataset	Calculated Measures		
	Accuracy (%)	Error (%)	Testing Time (s)
ISBI 2016	89.37	10.63	46.0923
ISBI 2017	90.46	9.54	51.4206
ISBI 2018	82.09	17.91	56.3782
PH2	91.30	8.7	23.5417

**Table 3:** Comparison of segmentation accuracy of the proposed method with existing methods.

Reference	Dataset	Segmentation Accuracy (%)
[61]	ISBI 2016	94.79
[61]	ISBI 2017	94.92
[61]	PH2	95.86
[62]	ISIC 2017	94.98
[62]	PH2	95.41
[63]	ISIC 2017	94.08
Proposed	ISBI 2016	95.38
	ISBI 2017	95.79
	ISBI 2018	92.69
	PH2	98.70

**Table 4:** Proposed multiple skin lesion type classification results using HAM10000 dataset. Best values are shown in bold.

Classifier	Performance Measures			
	Accuracy (%)	Sensitivity (%)	FNR (%)	Prediction Time (s)
Naïve Bayes	81.34	81.24	18.66	157.3042
ELM	84.92	84.90	15.08	138.5049
KELM	**90.67**	**90.20**	**9.33**	133.4406
MSVM	85.50	85.42	14.50	**121.5200**
Fine KNN	82.08	82.00	17.92	139.3896

**Table 5:** Confusion matrix of the KELM for multiple skin lesion type classification.

Actual Class	Predicted Skin Lesion Type						
	AKIEC	BCC	BKL	DF	NV	MEL	VASC
AKIEC	90.42%	2%		4%	2%		<2%
BCC		94.60%		5%		<1%	
BKL		<2%	93.04%		5%		
DF	2%		1%	84.30%	4%	7%	5%
NV		2%		2%	88.92%	<1%	7%
MEL	65	1%	2%			90.64%	<1%
VASC			5%		4%	1%	89.50%

**Table 6:** Multiple skin lesion type classification results using only the ResNet101 CNN model after transfer learning. Best values are shown in bold.

Classifier	Performance Measures		
	Accuracy (%)	FNR (%)	Prediction Time (s)
Naïve Bayes	73.64	26.36	171.6642
ELM	76.24	23.76	146.3290
KELM	**80.46**	**19.54**	149.5046
MSVM	77.50	22.5	**136.3604**
Fine KNN	74.94	25.06	148.9920

**Table 7:** Multiple skin lesion type classification results using only the DenseNet201 CNN model after transfer learning. Best values are shown in bold.

Classifier	Performance Measures		
	Accuracy (%)	FNR (%)	Prediction Time (s)
Naïve Bayes	75.36	24.64	172.6420
ELM	76.42	23.58	148.9260
KELM	**79.34**	**20.66**	145.3364
MSVM	78.16	21.84	**132.2064**
Fine KNN	74.30	25.7	145.3092

**Table 8:** Multiple skin lesion type classification results using optimal ResNet101 deep features. Best values are shown in bold.

Classifier	Performance Measures		
	Accuracy (%)	FNR (%)	Prediction Time (s)
Naïve Bayes	77.84	22.16	114.4534
ELM	78.36	21.64	101.5426
KELM	**83.04**	**16.96**	103.9962
MSVM	80.12	19.88	**96.3248**
Fine KNN	76.04	23.96	107.9040

**Table 9:** Multiple skin lesion type classification results using optimal DenseNet201 deep features. Best values are shown in bold.

Classifier	Performance Measures		
	Accuracy (%)	FNR (%)	Prediction Time (s)
Naïve Bayes	78.14	21.86	110.3044
ELM	80.29	19.71	96.5409
KELM	**84.04**	**15.96**	98.3667
MSVM	81.30	18.7	**90.2014**
Fine KNN	80.49	19.51	97.2436

**Table 10:** Comparison of the proposed method with the existing techniques.

Reference	Year	Dataset	Accuracy (%)	Sensitivity (%)
[64]	2020	HAM10000	83.0	83.0
[33]	2020	HAM10000	-	75.57
Proposed	2020	HAM10000	90.67	90.20

**Table 11:** Comparison of the proposed improved MFO with original MFO. Best values are shown in bold.

Method/ Optimization Technique	Evaluation Measures		
	Accuracy (%)	Sensitivity (%)	Error (%)
KELM/MFO	86.24	86.20	13.76
KELM/IMFO	**90.67**	**90.70**	**9.33**
Softmax/MFO	82.96	82.94	17.04
Softmax/IMFO	87.45	87.52	12.55

## Data Availability

The ISBI/ISIC challenge datasets are available at https://challenge.isic-archive.com/data (accessed on 28 April 2021). The PH2 dataset is available at https://www.fc.up.pt/addi/ph2%20database.html (accessed on 28 April 2021). The HAM10000 dataset is available at https://doi.org/10.7910/DVN/DBW86T (accessed on 28 April 2021).

## References

[1] Barata C., Celebi M.E., Marques J.S. (2021). Explainable skin lesion diagnosis using taxonomies. Pattern Recognit..

[2] Liu L., Tsui Y.Y., Mandal M. (2021). Skin lesion segmentation using deep learning with auxiliary task. J. Imaging.

[3] Mohapatra S., Abhishek N.V.S., Bardhan D., Ghosh A.A., Mohanty S. (2020). Skin cancer classification using convolution neural networks. Lecture Notes in Networks and Systems.

[4] Rogers H.W., Weinstock M.A., Feldman S.R., Coldiron B.M. (2015). Incidence Estimate of Nonmelanoma Skin Cancer (Keratinocyte Carcinomas) in the US Population, 2012. JAMA Dermatol..

[5] Guy G.P., Machlin S.R., Ekwueme D.U., Yabroff K.R. (2015). Prevalence and costs of skin cancer treatment in the U.S.; 2002−2006 and 2007−2011. Am. J. Prev. Med..

[6] American Cancer Society Annual Cancer Facts and Figures. https://www.cancer.org/content/dam/cancer-org/research/cancer-facts-and-statistics/annual-cancer-facts-and-figures/2020/cancer-facts-and-figures-2020.pdf.

[7] Siegel R.L., Miller K.D., Jemal A. (2019). Cancer statistics, 2019. CA Cancer J. Clin..

[8] Bandic J., Kovacevic S., Karabeg R., Bandic M., Lazarov A., Opric D. (2020). Teledermoscopy for skin cancer prevention: A comparative study of clinical and teledermoscopic diagnosis. Acta Inform. Med..

[9] Argenziano G., Fabbrocini G., Carli P., De Giorgi V., Sammarco E., Delfino M. (1998). Epiluminescence microscopy for the diagnosis of doubtful melanocytic skin lesions. Arch. Dermatol..

[10] Singh V.K., Abdel-Nasser M., Rashwan H.A., Akram F., Pandey N., Lalande A., Presles B., Romani S., Puig D. (2019). FCA-net: Adversarial learning for skin lesion segmentation based on multi-scale features and factorized channel attention. IEEE Access.

[11] Celebi M.E., Codella N., Halpern A. (2019). Dermoscopy image analysis: Overview and future directions. IEEE J. Biomed. Health Inform..

[12] Vestergaard M.E., Macaskill P., Holt P.E., Menzies S.W. (2008). Dermoscopy compared with naked eye examination for the diagnosis of primary melanoma: A meta-analysis of studies performed in a clinical setting. Br. J. Dermatol..

[13] Barata C., Celebi M.E., Marques J.S. (2019). A survey of feature extraction in dermoscopy image analysis of skin cancer. IEEE J. Biomed. Health Inform..

[14] Kassem M.A., Hosny K.M., Fouad M.M. (2020). Skin lesions classification into eight classes for ISIC 2019 using deep convolutional neural network and transfer learning. IEEE Access.

[15] Szaleniec J., Szaleniec M., Stręk P., Boroń A., Jabłońska K., Gawlik J., Składzień J. (2014). Outcome prediction in endoscopic surgery for chronic rhinosinusitis—A multidimensional model. Adv. Med. Sci..

[16] Piekarski M., Jaworek-Korjakowska J., Wawrzyniak A.I., Gorgon M. (2020). Convolutional neural network architecture for beam instabilities identification in Synchrotron Radiation Systems as an anomaly detection problem. Measurement.

[17] Nisa M., Shah J.H., Kanwal S., Raza M., Khan M.A., Damaševičius R., Blažauskas T. (2020). Hybrid malware classification method using segmentation-based fractal texture analysis and deep convolution neural network features. Appl. Sci..

[18] Wei Z., Song H., Chen L., Li Q., Han G. (2019). Attention-based DenseUnet network with adversarial training for skin lesion segmentation. IEEE Access.

[19] Khan M.A., Akram T., Sharif M., Javed K., Rashid M., Bukhari S.A.C. (2019). An integrated framework of skin lesion detection and recognition through saliency method and optimal deep neural network features selection. Neural Comput. Appl..

[20] Esteva A., Kuprel B., Novoa R.A., Ko J., Swetter S.M., Blau H.M., Thrun S. (2017). Dermatologist-level classification of skin cancer with deep neural networks. Nature.

[21] Glowacz A., Glowacz Z. (2016). Recognition of images of finger skin with application of histogram, image filtration and K-NN classifier. Biocybern. Biomed. Eng..

[22] Tang J., Alelyani S., Liu H. (2014). Feature selection for classification: A review. Data Classification: Algorithms and Applications.

[23] Tschandl P., Rosendahl C., Kittler H. (2018). The HAM10000 dataset, a large collection of multi-source dermatoscopic images of common pigmented skin lesions. Sci. Data.

[24] Gutman D., Codella N.C., Celebi E., Helba B., Marchetti M., Mishra N., Halpern A. (2016). Skin lesion analysis toward melanoma detection: A challenge at the international symposium on biomedical imaging (ISBI), hosted by the international skin imaging collaboration (ISIC). arXiv.

[25] Codella N.C., Gutman D., Celebi M.E., Helba B., Marchetti M.A., Dusza S.W., Kalloo A., Liopyris K., Mishra N., Kittler H. Skin lesion analysis toward melanoma detection: A challenge at the 2017 international symposium on biomedical imaging (ISBI), hosted by the international skin imaging collaboration (ISIC). Proceedings of the 2018 IEEE 15th International Symposium on Biomedical Imaging (ISBI 2018).

[26] Codella N., Rotemberg V., Tschandl P., Celebi M.E., Dusza S., Gutman D., Helba B., Kalloo A., Liopyris K., Marchetti M. (2019). Skin lesion analysis toward melanoma detection 2018: A challenge hosted by the international skin imaging collaboration (ISIC). arXiv.

[27] Mendonca T., Celebi M., Mendonca T., Marques J. (2015). Ph2: A public database for the analysis of dermoscopic images. Dermoscopy Image Analysis.

[28] He K., Zhang X., Ren S., Sun J. Deep residual learning for image recognition. Proceedings of the IEEE Conference on Computer Vision and Pattern Recognition.

[29] Huang G., Liu Z., Van Der Maaten L., Weinberger K.Q. Densely Connected Convolutional Networks. Proceedings of the IEEE Conference on Computer Vision and Pattern Recognition (CVPR).

[30] Khan M.A., Akram T., Sharif M., Shahzad A., Aurangzeb K., Alhussein M., Haider S.I., Altamrah A. (2018). An implementation of normal distribution based segmentation and entropy controlled features selection for skin lesion detection and classification. BMC Cancer.

[31] Tschandl P., Sinz C., Kittler H. (2019). Domain-specific classification-pretrained fully convolutional network encoders for skin lesion segmentation. Comput. Biol. Med..

[32] Mahbod A., Schaefer G., Wang C., Dorffner G., Ecker R., Ellinger I. (2020). Transfer learning using a multi-scale and multi-network ensemble for skin lesion classification. Comput. Methods Programs Biomed..

[33] Harangi B., Baran A., Hajdu A. (2020). Assisted deep learning framework for multi-class skin lesion classification considering a binary classification support. Biomed. Signal Process. Control.

[34] Chaturvedi S.S., Tembhurne J.V., Diwan T. (2020). A multi-class skin Cancer classification using deep convolutional neural networks. Multimed. Tools Appl..

[35] Al-masni M.A., Kim D.-H., Kim T.-S. (2020). Multiple skin lesions diagnostics via integrated deep convolutional networks for segmentation and classification. Comput. Methods Programs Biomed..

[36] Xie Y., Zhang J., Xia Y., Shen C. (2020). A mutual bootstrapping model for automated skin lesion segmentation and classification. IEEE Trans. Med. Imaging.

[37] Jayapriya K., Jacob I.J. (2019). Hybrid fully convolutional networks-based skin lesion segmentation and melanoma detection using deep feature. Int. J. Imaging Syst. Technol..

[38] Russakovsky O., Deng J., Su H., Krause J., Satheesh S., Ma S., Huang Z., Karpathy A., Khosla A., Bernstein M. (2015). ImageNet large scale visual recognition challenge. Int. J. Comput. Vis..

[39] Szegedy C., Liu W., Jia Y., Sermanet P., Reed S., Anguelov D., Erhan D., Vanhoucke V., Rabinovich A. Going deeper with convolutions. Proceedings of the 2015 IEEE Conference on Computer Vision and Pattern Recognition (CVPR).

[40] Xie F., Yang J., Liu J., Jiang Z., Zheng Y., Wang Y. (2020). Skin lesion segmentation using high-resolution convolutional neural network. Comput. Methods Programs Biomed..

[41] Miglani V., Bhatia M. Skin lesion classification: A transfer learning approach using efficientnets. Proceedings of the International Conference on Advanced Machine Learning Technologies and Applications (AMLTA 2020).

[42] Mahbod A., Schaefer G., Ellinger I., Ecker R., Pitiot A., Wang C. (2019). Fusing fine-tuned deep features for skin lesion classification. Comput. Med. Imaging Graph..

[43] Mahbod A., Tschandl P., Langs G., Ecker R., Ellinger I. (2020). The effects of skin lesion segmentation on the performance of dermatoscopic image classification. Comput. Methods Programs Biomed..

[44] Kim Y.-T. (1997). Contrast enhancement using brightness preserving bi-histogram equalization. IEEE Trans. Consum. Electron..

[45] Akram T., Khan M.A., Sharif M., Yasmin M. (2018). Skin lesion segmentation and recognition using multichannel saliency estimation and M-SVM on selected serially fused features. J. Ambient Intell. Humaniz. Comput..

[46] Ahn E., Kim J., Bi L., Kumar A., Li C., Fulham M., Feng D.D. (2017). Saliency-Based Lesion Segmentation via Background Detection in Dermoscopic Images. IEEE J. Biomed. Health Inform..

[47] Awan M.J., Mohd Rahim M.S., Salim N., Mohammed M.A., Garcia-Zapirain B., Abdulkareem K.H. (2021). Efficient detection of knee anterior cruciate ligament from magnetic resonance imaging using deep learning approach. Diagnostics.

[48] Tu W., Liu X., Hu W., Pan Z. (2019). Dense-residual network with adversarial learning for skin lesion segmentation. IEEE Access.

[49] Fan H., Xie F., Li Y., Jiang Z., Liu J. (2017). Automatic segmentation of dermoscopy images using saliency combined with Otsu threshold. Comput. Biol. Med..

[50] Achanta R., Shaji A., Smith K., Lucchi A., Fua P., Süsstrunk S. (2012). SLIC Superpixels Compared to State-of-the-Art Superpixel Methods. IEEE Trans. Pattern Anal. Mach. Intell..

[51] Wang S.-H., Zhang Y.-D. (2020). DenseNet-201-Based Deep Neural Network with Composite Learning Factor and Precomputation for Multiple Sclerosis Classification. ACM Trans. Multimed. Comput. Commun. Appl..

[52] Zhuang F., Qi Z., Duan K., Xi D., Zhu Y., Zhu H., Xiong H., He Q. (2021). A comprehensive survey on transfer learning. Proc. IEEE.

[53] Urbonas A., Raudonis V., Maskeliunas R., Damaševičius R. (2019). Automated identification of wood veneer surface defects using faster region-based convolutional neural network with data augmentation and transfer learning. Appl. Sci..

[54] Nanni L., Interlenghi M., Brahnam S., Salvatore C., Papa S., Nemni R., Castiglioni I. (2020). Comparison of transfer learning and conventional machine learning applied to structural brain MRI for the early diagnosis and prognosis of alzheimer’s disease. Front. Neurol..

[55] Rehman Z.U., Khan M.A., Ahmed F., Damaševičius R., Naqvi S.R., Nisar W., Javed K. (2021). Recognizing apple leaf diseases using a novel parallel real-time processing framework based on MASK RCNN and transfer learning: An application for smart agriculture. IET Image Process..

[56] Khan M.A., Ashraf I., Alhaisoni M., Damaševičius R., Scherer R., Rehman A., Bukhari S.A.C. (2020). Multimodal brain tumor classification using deep learning and robust feature selection: A machine learning application for radiologists. Diagnostics.

[57] Elaziz M.A., Ewees A.A., Ibrahim R.A., Lu S. (2020). Opposition-based moth-flame optimization improved by differential evolution for feature selection. Math. Comput. Simul..

[58] Mirjalili S. (2015). Moth-flame optimization algorithm: A novel nature-inspired heuristic paradigm. Knowl. Based Syst..

[59] Jaeschke D. (1979). The asymptotic distribution of the supremum of the standardized empirical distribution function on subintervals. Ann. Stat..

[60] Liu X., Wang L., Huang G.-B., Zhang J., Yin J. (2015). Multiple kernel extreme learning machine. Neurocomputing.

[61] Rehman A., Khan M.A., Mehmood Z., Saba T., Sardaraz M., Rashid M. (2020). Microscopic melanoma detection and classification: A framework of pixel-based fusion and multilevel features reduction. Microsc. Res. Tech..

[62] Saba T., Khan M.A., Rehman A., Marie-Sainte S.L. (2019). Region extraction and classification of skin cancer: A heterogeneous framework of deep CNN features fusion and reduction. J. Med. Syst..

[63] Bi L., Kim J., Ahn E., Kumar A., Feng D., Fulham M. (2019). Step-wise integration of deep class-specific learning for dermoscopic image segmentation. Pattern Recognit..

[64] Chaturvedi S.S., Gupta K., Prasad P.S. (2020). Skin lesion analyser: An efficient seven-way multi-class skin cancer classification using mobilenet. Advances in Intelligent Systems and Computing.

